# Osteopontin and malaria: no direct effect on parasite growth, but correlation with *P. falciparum-*specific B cells and BAFF in a malaria endemic area

**DOI:** 10.1186/s12866-021-02368-y

**Published:** 2021-11-06

**Authors:** Susanne E Mortazavi, Allan Lugaajju, Mark Kaddumukasa, Muyideen Kolapo Tijani, Fred Kironde, Kristina E M Persson

**Affiliations:** 1 Department of Laboratory Medicine, Lund University, Skåne University Hospital, Lund, Sweden; 2 Department of Infectious Diseases, Skåne University Hospital, Lund, Sweden; 3 College of Health Sciences, Makerere University, Kampala, Uganda; 4 Cellular Parasitology Program, Cell Biology and Genetics Unit, Department of Zoology, University of Ibadan, Ibadan, Nigeria; 5 Habib Medical School, Faculty of Health Sciences, Islamic University in Uganda, Kampala, Uganda

**Keywords:** Osteopontin, Malaria, *Plasmodium falciparum*, B cell, BAFF, Immunity

## Abstract

**Background:**

The dysregulation of B cell activation is prevalent during naturally acquired immunity against malaria. Osteopontin (OPN), a protein produced by various cells including B cells, is a phosphorylated glycoprotein that participates in immune regulation and has been suggested to be involved in the immune response against malaria. Here we studied the longitudinal concentrations of OPN in infants and their mothers living in Uganda, and how OPN concentrations correlated with B cell subsets specific for *P. falciparum* and B cell activating factor (BAFF). We also investigated the direct effect of OPN on *P. falciparum in vitro*.

**Results:**

The OPN concentration was higher in the infants compared to the mothers, and OPN concentration in infants decreased from birth until 9 months. OPN concentration in infants during 9 months were independent of OPN concentrations in corresponding mothers. OPN concentrations in infants were inversely correlated with total atypical memory B cells (MBCs) as well as *P. falciparum-*specific atypical MBCs. There was a positive correlation between OPN and BAFF concentrations in both mothers and infants. When OPN was added to *P. falciparum* cultured *in vitro*, parasitemia was unaffected regardless of OPN concentration.

**Conclusions:**

The concentrations of OPN in infants were higher and independent of the OPN concentrations in corresponding mothers. *In vitro*, OPN does not have a direct effect on *P. falciparum* growth. Our correlation analysis results suggest that OPN could have a role in the B cell immune response and acquisition of natural immunity against malaria.

**Supplementary Information:**

The online version contains supplementary material available at 10.1186/s12866-021-02368-y.

## Background

*P. falciparum* malaria still remains a major health threat in sub-Saharan Africa, and children aged less than 5 years of age and pregnant women are particularly vulnerable. In 2019, there was an estimated 409 000 deaths from malaria globally of which 94 % occurred in the African region [1]. Malaria in pregnancy represents a special challenge and is associated with low birth weight, maternal anemia, preterm delivery and stillbirth [2–4]. *P. falciparum* infected red blood cells (RBC) expressing the parasite-derived protein VAR2CSA sequester in the placenta, which can partly explain the increased susceptibility to malaria in pregnant women [5].

Although sterile immunity against malaria is almost never achieved, individuals living in malaria endemic areas can acquire clinical immunity to severe forms of *P. falciparum* malaria [6, 7]. To date, there is no effective malaria vaccine that can function in a non-immune population, and the mechanisms underlying the development of immunity are not well defined. However, naturally acquired immunity against blood stage parasites has been shown to involve both CD4+ T cells and antibodies [8, 9]. Studies have demonstrated that passively transferred IgG from semi-immune adults with repeated prior exposure to *P. falciparum* can clear or reduce parasitemia in children acutely infected with *P. falciparum* [10]. Numerous investigations have revealed that the quality, level, and breadth of the antibody response are important components of clinical immunity in malaria [11–14]. In addition, primigravidae are at highest risk of *P. falciparum* pregnancy-associated malaria compared to multigravidae, and multiple pregnancies lead to the acquisition of antibodies against VAR2CSA, which reduces the prevalence and severity of infection [15, 16].

The slow development of malaria immunity and the poor sustainability suggest an interference with the immune homeostasis by the malaria parasite [17]. In order to understand the production and sustainability of antibodies, B cell studies are needed. For malaria, alterations such as polyclonal B cell activation, atypical memory B cell expansion, and deletion of specific B cell subsets are well described [17–23]. Nevertheless, the mechanisms leading to this B cell dysregulation are not entirely understood.

Osteopontin (OPN) is a phosphorylated glycophosphoprotein also referred to as early T-lymphocyte activation-1 (Eta-1) factor or secreted phosphoprotein-1 (SPP-1) [24]. Osteopontin is involved in numerous biological functions depending on its intra- or extracellular localization, such as bone mineralization, wound healing, inflammatory diseases, cancer, cellular adhesion and migration [25–27], as well as immune regulation [28]. It is expressed in various tissues and cells, including immune cells such as neutrophils, macrophages and B- and T cells [29]. Osteopontin plays an important role in the Th1 immune response. *In vivo*, mice deficient in *OPN* gene expression were reported to have severely compromised type-1 immunity to some intracellular infections such as *Listeria monocytogenes*, *Mycobacterium bovis* and *Herpes simplex virus type 1*, potentially due to decreased production of IL-12 and interferon-gamma [30]. Besides having a key role in dendritic cell maturation and migration [26], OPN additionally functions as a polyclonal B cell activator and stimulates immunoglobulin production, particularly IgM and IgG3 antibodies [31, 32]. Increased OPN concentration have also been reported to be associated with diseases of autoimmune B cell involvement such as systemic lupus erythematosus [33], psoriasis [34] and Sjögren’s disease [35]. In severe infections, OPN is believed to be expressed by B cells, as IL-4 activates B cell signaling through BCR and PI3K, NF-κB and pERK [36].

Studies indicate that members of the tumor necrosis factor (TNF) superfamily such as B cell activation factor (BAFF; also known as BlyS) and a proliferation-inducing ligand (APRIL), have important roles in T cell independent antibody production, immunoglobulin isotype switching and in the selection, maturation and survival of B cells [37, 38]. In a mouse model with multiple sclerosis, it was demonstrated that BAFF-stimulated B cells increased OPN secretion, leading to expression of the anti-apoptotic molecule Bcl2 in T cells [39]. These results suggest a role for both BAFF and OPN in T cell survival in autoimmune diseases.

To our knowledge there are merely two previous reports on the potential role of OPN in malaria infection. In a Vietnamese cohort positive for *P. falciparum*, OPN mRNA was detected in a majority of cases, and in these there was also a higher expression of both interleukin-12 p40 and interferon-γ [40]. The level of parasitemia was lower in the OPN mRNA-positive group, suggesting OPN has a role in suppressing multiplication of parasites. Correspondingly, the second study demonstrated how OPN knock-out mice died of the murine malaria *P. chaubadi chaubadi*, while wild-type mice had self-limiting infections [41].

Previous studies of OPN concentrations in cord blood are sparse, and to our knowledge no other study has investigated OPN concentration in both infants and their mothers in a malaria endemic area. In the present study, we measured longitudinal variations in concentrations of OPN in mothers and their infants and monitored them over nine months. We also determined correlations between concentrations of OPN and *P. falciparum*-specific B cell subsets, and antibodies to *P. falciparum* schizont extract as well as concentrations of BAFF [21, 42]. Additionally, we investigated the effect of OPN on parasitic growth using an *in vitro* invasion assay. These results are important for understanding acquisition of natural immunity to malaria, and for further vaccine and treatment studies of the disease.

## Results

### Characteristics of participants

Blood samples from 80 mother-infant pairs were included in the analysis of OPN concentration. Fifty-eight pairs had samples from all six time points: mothers at birth, infants at birth, infants at 2.5, 6 and 9 months and mothers at 9 months. Only 18 pairs had sufficient sample material for five of the six time points and 4 pairs had enough for four time points. Plasma samples collected from anonymous 35 Ugandan and 20 Swedish healthy male adults were included as controls.

### Concentration of OPN in plasma in mothers and infants

**Fig. 1:**
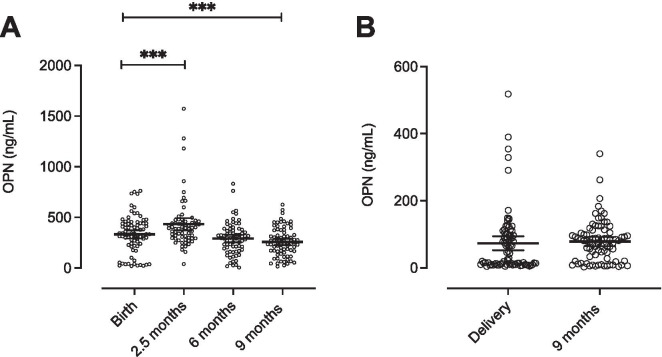
Distribution of individual and estimated mean OPN plasma concentrations (ng/mL) in infants and mothers at each time point. Each dot represents an individual value, horizontal lines the estimated mean from linear mixed models, and whiskers show the corresponding 95 % confidence intervals; *** significant at P < 0.001 tested by linear mixed models. **A** OPN concentrations in infants at birth, 2.5 months, 6 months, and 9 months. **B** OPN concentrations in mothers at delivery and after 9 months

Individual concentrations of OPN in infants varied between 5 ng/mL (at 6 months of age) and 1573 ng/mL (at 2.5 months) (Fig. 1A). In the mothers, the lowest and highest individual concentrations were 3 and 518 ng/mL, respectively (Fig. 1B), measured at the 9-month follow-up and at delivery. Estimated mean OPN concentrations from linear mixed models are shown in Fig. 1 A and B. The estimated mean concentration in cord blood was 332 (95 % CI, 292-373) ng/mL, in infants at 2.5 months 433 (95 % CI, 388-478) ng/mL, in infants at 6 months 292 (95 % CI, 249-335) ng/mL, in infants at 9 months 258 (95 % CI, 216-300) ng/mL, in mothers at delivery 73 (95 % CI, 56-91) ng/mL, and in mothers at 9 months 78 (95 % CI, 61-96) ng/mL.

Linear mixed models were performed to adjust for the intra-individual dependence in the analysis of a potential change in estimated mean concentration of OPN at the different time points. Estimated mean OPN concentration in the infants significantly increased between birth and 2.5 months (P < 0.001); 101 ng/mL (confidence interval 41; 161) and significantly decreased between birth and 9 months (P < 0.001); -74 ng/mL (-132; -17) (Fig. 1A). Adjusting for the mothers’ values in the linear mixed models had no statistical effect on the infants’ estimated mean OPN concentration. There was no significant change in estimated mean concentrations of OPN in the mothers from delivery to 9 months after delivery as analyzed by linear mixed models (Fig. 1B).

**Fig. 2:**
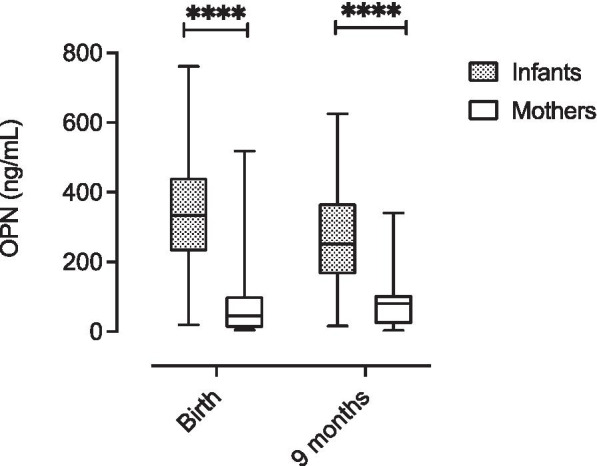
Distribution of OPN plasma concentrations in infants and their mothers at birth and after 9 months. Box plots represent interquartile range, whiskers the range and horizontal lines represent the median; **** significant at P < 0.0001 tested by Wilcoxon matched-pairs signed-rank test

To specifically investigate the differences between infants and mothers, Fig. 2 shows that the OPN concentrations were significantly higher in infants compared to the mothers at birth, as well as after nine months (Wilcoxon matched-pairs signed-rank test was used for this comparison).

#### Osteopontin plasma concentration in control groups

**Fig. 3:**
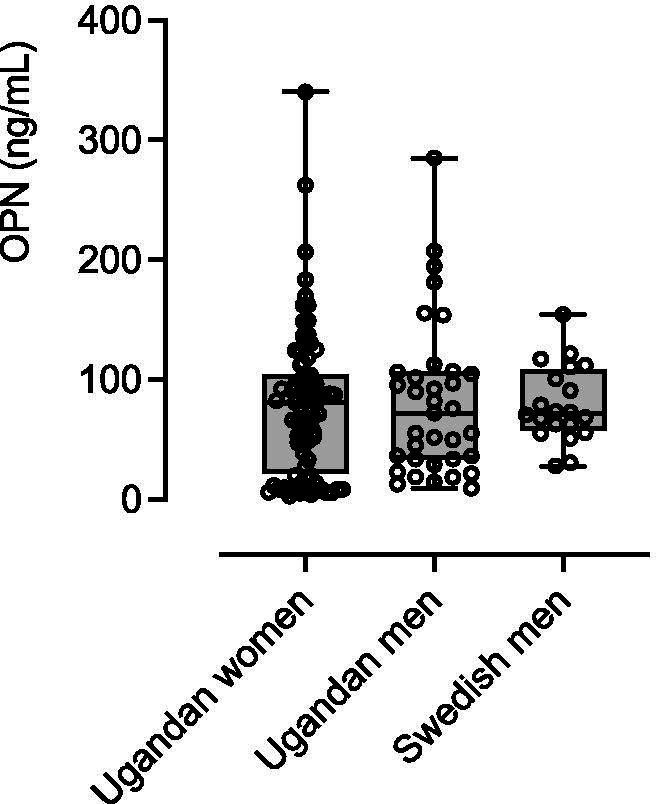
Distribution of individual and median OPN plasma concentrations (ng/mL) in 35 healthy adult Ugandan men, 80 Ugandan women and 20 men from Sweden. The scatterplot shows the individual values, box plots represent the interquartile range (IQR), the horizontal lines the median, and whiskers the minimum and maximum. There was no significant difference between the groups as tested by Kruskal-Wallis test

We analyzed OPN concentrations in 35 and 20 healthy men in Uganda and Sweden, respectively. There were varying concentrations of OPN in both groups, particularly in the Ugandan group with OPN concentrations varying between 9 and 285 ng/mL. Median OPN concentrations in the Ugandan group was 72 (IQR = 107-34) ng/mL and in the Swedish group it was 72 (IQR = 108-57) ng/mL, and there was no significant difference in OPN concentration between the two groups (Fig. 3). Sex analysis of differences of OPN concentrations was performed in the Ugandan cohort (Fig. 3) and there was no significant difference between women and men.

### Osteopontin concentration and *P. falciparum* parasitemia

Nine individuals tested positive for *P. falciparum* with rapid diagnostic test (RDT) and microscopy during our study period. We did not observe any difference in OPN concentration subject to the degree of parasitemia (Additional file 1), and we concluded that the sample size was not sufficient to show any potential statistical differences.

### Correlation between *P. falciparum* schizont-specific antibodies and OPN concentrations

As previously reported, antibody levels to schizont extract in all individuals were analyzed [21]. The mothers had no differences in levels of schizont specific IgG or IgM antibodies between the two time points. As expected, the infants at birth showed higher levels of IgG directed against *P. falciparum* (presumably transferred through the placenta), while the levels decreased at 2.5 months to be followed by a gradual increase of parasite specific IgG and IgM during the last part of the study period, as they became exposed to malaria [21]. In the present study, we correlated the antibody levels with OPN concentrations using Spearman’s correlation to examine possible associations. The data was compared individually for each time point. The correlation analysis for OPN and antibodies to schizont extract generated no significant associations between OPN concentrations and IgG or IgM in infants (not shown). In mothers at delivery and after nine months there was a significant correlation between OPN and schizont specific IgG (r = 0.29 P = 0.016, and r = 0.35 P = 0.003), but not between OPN and IgM.

### Correlation between total and *P. falciparum*-positive (*Pf*+) B cell subsets and plasma OPN concentrations

**Fig. 4:**
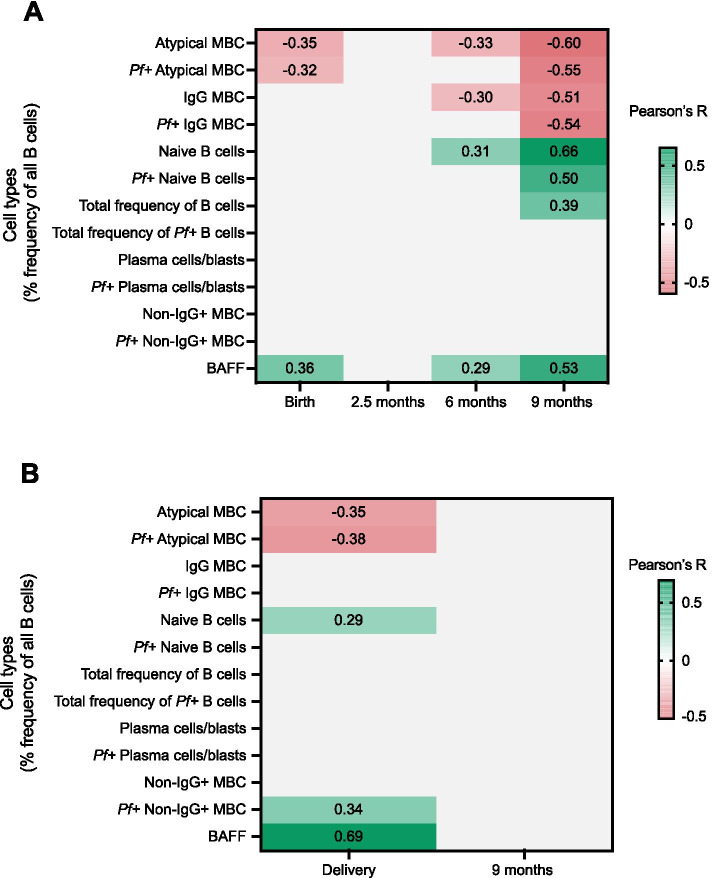
Correlation heatmap of OPN concentration and subsets of B cells and BAFF in infants and mothers. Pearson correlation with adjustment for multiple comparisons using the Benjamini-Hochberg method performed for each time point, presenting R values with P <0.05. Green color represents positive correlations and red color represents negative correlations. **A** OPN correlation heatmap for infants at birth, 2.5 months, 6 months, and 9 months. **B** OPN correlation heatmap for mothers at delivery and after 9 months

To further study the role of OPN in B cell activities we employed a correlation analysis using Pearson’s correlation test and adjustment for multiple comparisons with the Benjamini-Hochberg method for each time point, and separately for mothers and infants. The analysis assessed correlations between OPN and mean proportions of *Pf+* B cell subsets and total B cells that have been previously described [21]. For infants at birth there were significant negative correlations in OPN concentration and atypical MBC (r = -0.35, P = 0.01) and *Pf+* atypical MBC (r = -0.32, P = 0.021) (Fig. 4A). In infants at 6 months, there were significant negative correlations in OPN concentration and atypical MBC (r = -0.33, P = 0.021) and IgG MBC (r = -0.30, P = 0.038). Inversely, OPN concentration were positively correlated with naïve B cells (r = 0.31, P = 0.033). OPN concentration in infants at 9 months were negatively correlated with IgG MBC (r = -0.51, P <0.001) and atypical MBC (r = -0.60, P <0.001), including *Pf+* proportions for both these compartments (r= -0.54, P <0.001 and r = -0.55, P <0.001. At the same time point, positive correlations in OPN concentration were found for naïve B cells (r = 0.66, P <0.001), *Pf+* naïve B cells (r = 0.50, P <0.001) and total frequency of B cells (r = 0.39, P = 0.004) (Fig. 4A). In mothers at delivery, OPN concentration were negatively correlated with atypical MBC (r = -0.35, P = 0.011) and *Pf+* atypical MBC (r = -0.38, P = 0.006). On the other hand, OPN concentration were positively correlated with naïve B cells (r = 0.29, P = 0.041) and *Pf+* non-IgG+ MBC (r = 0.34, P = 0.013) (Fig. 4B). X-Y scatter plots of variables with significant correlations are shown in Additional file 2.

### Correlation between plasma concentrations of BAFF and OPN

Since both BAFF and OPN have roles in B cell differentiation, we compared OPN concentrations with BAFF concentrations, recently reported for these individuals [42]. OPN concentration in infants were positively correlated with BAFF concentrations at birth (r= 0.36, P = 0.003), 6 months (r= 0.29, P = 0.035) and 9 months (r= 0.53, P <0.001) (Fig. 4A). Likewise, in mothers at delivery, OPN concentration were positively correlated with BAFF concentration (r= 0.69, P <0.001) (Fig. 4B). X-Y scatter plots of variables with significant correlations are shown in Additional file 2.

### Effect of Osteopontin on *P. falciparum* invasion *in vitro*

**Fig. 5:**
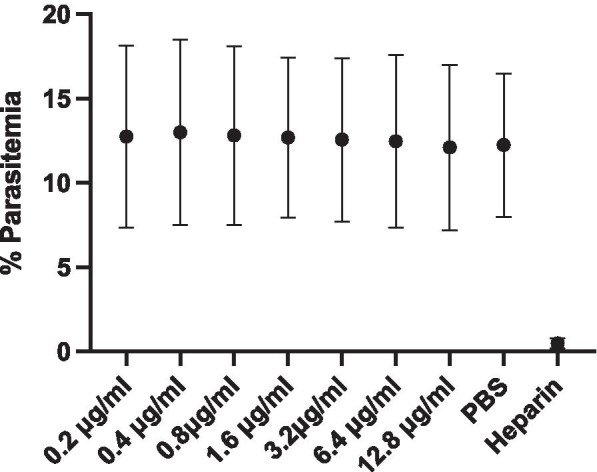
*P. falciparum* invasion inhibition assay performed over two life cycles. Mean parasitemia obtained from two repeated assays (each performed in duplicate) with different concentrations of OPN (ranging from 200 ng/mL to 12 800 ng/mL) added to FCR3S1.2 parasites. There was no statistically significant difference between the mean parasitemias when OPN was added in the different concentrations. PBS and heparin (100 µg/mL) were added as controls

We added recombinant OPN to FCR3S1.2 *P. falciparum* cultures. At first, we added OPN at different concentrations in assays for one life cycle (not shown), but since we did not see any effect, we increased the time for the experiments to 90 h as described in [43]. We could not see any significant differences in parasitemia levels even at concentrations above what is known to be physiological (Fig. 5). PBS was included as a control for growth, and heparin was used as a negative control since it is a known inhibitor of *P. falciparum* parasites.

## Discussion

This is the first report of osteopontin longitudinal measurements in a cohort of healthy mother-infant pairs from birth up to nine months. This is also the first description of OPN values in mothers and infants in a malaria-endemic setting. In general, the concentrations of OPN in the infants were higher than in the mothers. We were also able to demonstrate that estimated mean OPN concentrations in infants increased from birth to 2.5 months, perhaps due to exposure to pathogens after birth, but by 9 months it had decreased again. By using linear mixed model analyses we can report that OPN concentrations in infants from delivery to 9 months afterwards were independent of the OPN concentration in corresponding mothers. In addition, our data show stable OPN concentrations in mothers at delivery and after 9 months. These results suggest that OPN has a natural role in fetal development, as well as during the first months after birth, possibly reflecting a maturing immune system. Interestingly, the lowest and the highest values of OPN in infants and the mothers did not correspond within the same mother-infant pair. The OPN concentrations and variations in OPN concentration in infants did not change when linear mixed models were used to adjust for OPN concentrations in corresponding mothers, supporting the conclusion that the source of OPN in cord blood is either the fetus itself or that it is produced in the placenta [44], and that OPN is not transferred from the mother. Since there was no difference in OPN concentrations in the mothers between delivery and 9 months, this indicates that normal pregnancies do not induce any major changes in production of OPN, even in an area where malaria and several other infectious diseases are relatively common [45, 46].

In our study, mean OPN plasma concentrations in cord blood and in mothers at delivery were 332 ng/mL and 73 ng/mL respectively, concentrations comparable to studies performed in non-endemic countries [44, 47–49]. Similar to our results, previous reports have presented increased mean OPN concentrations in infants compared to adults [44, 48, 50] and no significant difference between pregnant and non-pregnant women [47, 48]. A prospective study aiming to establish OPN reference values in different age groups in Germany, demonstrated that OPN values in plasma were age dependent [50]. However, the mean OPN plasma concentration in cord blood and in adults were markedly higher (2300 ± 552 ng/mL and 330 ng/mL) compared to our results. Such a discrepancy in results between studies could be due to the use of OPN ELISA kits from different manufacturers, or because of different ethnicities in the study populations. Studies using ELISA kits from the same manufacturer as in this study present OPN plasma concentrations in healthy adults similar to ours [49, 51, 52]. Additionally, we included a small set of Swedish samples, and these results indicate that there is no difference in OPN concentration depending on ethnicity/country of residence or sex, but the cohort was small and not aged-matched, hence the results must be interpreted with caution. OPN concentrations have previously been reported to differ depending on sex and ethnicity [53], but the contrary has also been shown [54, 55]. Comparing the results of our mother-infant cohort with OPN concentrations from various regions in the world, ethnicity is not a likely reason for extreme differences in concentration [44, 47–49, 51].

The comparison of OPN between studies can be challenging also from the point of view that OPN concentrations can differ depending on if the blood sample analyzed was serum or plasma, and, for plasma, if the anticoagulant was EDTA, heparin or citrate [56]. When examining the results, it is important to consider that secreted OPN undergoes post-translational modifications and cleavage by thrombin and metalloproteinases, and it can circulate both as cleaved and full-length OPN [57]. In this present study we used plasma from EDTA supplemented blood, and we measured full-length OPN in the ELISA assay.

Interestingly, in our cohort there was a statistically significant increase in estimated mean OPN concentrations in infants from birth to 2.5 months of age. The difference indicates that infants have lower concentrations of OPN at birth. In theory, the OPN concentration peak after 2.5 months could perhaps be part of an immune response as the infants are subject to new pathogens. An alternate explanation is that OPN might be higher because of increased post-natal bone formation [58].

As previously described in detail, only a few individuals in the mother-infant cohort had a positive RDT [21]. We estimated the number (9 included in our study) to be insufficient for determining a statistical difference regarding the relation of OPN and malaria, but by looking at the data we can see that there is no obvious correlation between the very varying parasitemias and OPN concentrations (Additional file 1). An earlier study of *P. falciparum* positive individuals suggested OPN to have a role in the immune response against malaria in suppression of parasitemia [40]. The patients that were positive for OPN mRNA had lower parasitemias compared to patients negative for OPN mRNA, and the authors found elevated expressions of IL-12 and suggested that OPN might have a role in Th1-mediated immune responses. Since there is a lack of knowledge in this area, we wanted to study if the protein OPN could have a direct effect on the parasite in an environment lacking immune cells and other immunomodulatory factors. To study the possible inhibitory effect of OPN on *P. falciparum* parasites, we performed invasion inhibition assays with cultured *P. falciparum* parasites. When we did not see any changes after 48 h of parasite culture, we repeated the experiment, but extended the cultures to 90 h. As previously discussed, there is no real consensus on the physiological concentrations of OPN in plasma, and studies have shown a wide variety of both low and high plasma concentrations with increased concentrations in a number of diseases [51, 52, 59, 60]. For this reason, we chose to add OPN concentrations varying between 200 ng/mL and 12 800 ng/mL in our invasion assay, to be sure to add at least what could be physiological. None of the assays demonstrated suppression of parasite levels regardless of OPN concentration, even when the 90 h assay was repeated, proposing that OPN alone *in vitro* does not affect the life cycle of *P. falciparum* parasites. Nevertheless, it is possible that OPN could have a secondary effect on the parasites *in vivo* by interacting with host immune cells, cytokines or molecules.

Since none of our study individuals positive for *P. falciparum* by RDT and microscopy presented with malaria symptoms, these are most likely asymptomatic carriers. This taken into account, one could theorize that OPN does not increase or decrease in the presence of parasites alone, but that it needs to be accompanied by symptoms such as in a febrile malaria infection. Supporting this theory, a study of OPN in acute human *Schistosoma mansoni* infection demonstrated an increase in serum OPN concentrations, compared to OPN concentrations in the chronic hepatosplenic form of the disease [52]. Also, besides malaria and schistosomiasis, other infectious diseases characterized by involvement of chronic immune activation are HIV and tuberculosis. Elevated concentrations of OPN have been reported in lymph nodes and plasma in HIV-positive individuals [60–62], as well as in plasma of acute tuberculosis patients, correlating to the severity of pulmonary lesions [63–65]. Interestingly, the concentration of OPN was higher in patients with active pulmonary TB compared to latent tuberculosis [65, 66]. Considering the similarities between malaria, HIV and TB regarding chronic immune activation, one could hypothesize that OPN plays a specific role in the immunopathogenesis of these diseases.

One of the inclusion criteria to the study was normal pregnancies and deliveries, and none of the study individuals showed overt signs of illness during the follow-up period. Nonetheless we do not know if there were some individuals with unknown diseases such as auto-immune disorders, cancer or heart- or liver disease that are known to influence OPN concentrations in plasma [67–70]. Additionally, it is possible to imagine they could be infected with other parasitic diseases such as helminths that may affect the OPN concentrations. This could explain why some of the study individuals had markedly higher OPN values.

One major finding in this study is that mean OPN concentrations in mothers at delivery and mean OPN concentrations in infants, at several time points, were inversely correlated with atypical MBCs and *Pf+* atypical MBCs. Furthermore, we could demonstrate a positive correlation between mean OPN and BAFF concentrations in both mothers and infants, an association that appeared stronger as the infants grew up (Fig. 4A). The parasite specific antibody characteristics of the mother-infant cohort was presented in the report by Lugaajju *et al*, demonstrating the presence of parasite specific IgG in cord blood, transferred through the placenta, followed by a decrease and then an increase over time as the infants were exposed to malaria [21]. During the follow-up, infant parasite specific IgM levels subsequently increased, possibly mirroring the infant’s own malaria immune response. Additionally, the proportion of CD19+Pf+ B cells showed stable levels in mothers at delivery compared to 9 months later, indicating previous and constant exposure to malaria. For the infants, the CD19+*Pf+* B cells proportions were continuously lower than in their mothers’, suggesting full immunity is not achieved at 9 months of age. Taken together, this affirms that people living in the area are subject to *P. falciparum* exposure and the results reflect the infants’ primary immune responses during the first nine months of life. Atypical MBCs, classified for these individuals as CD19+CD20+CD27−IgG+, represented a considerable proportion of the B cell population [21]. Acquisition of atypical MBCs correlates with exposure to malaria as well as with increasing age [71–73], and in our study cohort these cells increased with age in the infants. It is suggested that chronic persisting infection with *P. falciparum* may drive the expansion of atypical MBCs in peripheral blood, but to this date it is not clear whether they have a protective or pathogenic role in the immune response against malaria [71, 72]. Here, in the infants, significant negative correlations were found between OPN concentrations and atypical MBCs and between OPN concentrations and *Pf+* atypical MBCs at three and two time points, respectively. There was also a significant negative correlation between OPN concentrations and total IgG MBC at 6 and 9 months, as well as for *Pf+* IgG MBCs at 9 months. Furthermore, positive correlations were seen between OPN and naïve and *Pf+* naïve B cells at 6 and 9 months. The correlations between these parameters increased as the infants aged. These data could reflect a maturing immune response in a malaria-endemic setting where OPN increases in the presence of or by the naïve B cells, and consecutively decreases as the B cell proportion matures into malaria specific memory cells and activated B cells such as atypical MBCs and IgG MBC. Since it is still unknown whether atypical MBCs have a protective role in malaria and previous studies have shown a susceptibility to infectious diseases when OPN is deficient [30, 41], the correlation between the two is quite intriguing. These data suggest that OPN could have a role in the B cell immune response to *P. falciparum* infection as well as in the formation of atypical MBCs. For mothers, OPN results correlated with atypical MBCs and *Pf+* atypical MBCs at delivery, but not after 9 months. Most mothers in the cohort received IPT during their pregnancies, leading to absence of parasitemia, which might at least in theory affect the proportion between OPN concentration and the level of atypical MBCs. There was a significant correlation between schizont specific IgG and OPN in mothers at delivery and after 9 months which could indicate that OPN is an important factor in upholding the immune response in individuals with continuous exposure to *P. falciparum*. Furthermore, in both mothers and infants, we found a positive correlation between OPN and BAFF during delivery and throughout the entire study period, which indicates that there could be a link between the two. BAFF has a crucial role in B cell differentiation and antibody production, and similar to OPN, is known to be overexpressed in autoimmune diseases [74, 75]. High concentrations of BAFF in plasma have been seen in patients with an acute malaria infection [76, 77] as well as in patients with viral and bacterial infections [78]. The results from our study support the idea that BAFF might influence OPN expression presented by Ma *et al* [39]. Their mouse model study demonstrated how BAFF regulated T cell survival by inducing OPN expression in B cells via a natural factor (NF)-kB dependent signaling pathway. Interestingly, associations between OPN and BAFF have been reported in patients with autoimmune thyroid disease, but not in the healthy control group, indicating a BAFF-OPN signal promoting disease progression [79]. On the contrary, as mentioned before, all women and infants in our cohort were healthy and had quite strong correlations between OPN and BAFF. However, considering how several studies have shown higher number of cells similar to atypical MBCs in autoimmune disorders [80], one can hypothesize that a BAFF-OPN interplay is of importance in the development of atypical MBCs in general and not only in a malaria context. Based on the results from this study, we believe it would be of further interest to study the correlations between OPN, BAFF and *Pf+* atypical MBCs to gain a more detailed understanding of the mechanisms of the formation and function of atypical MBCs in a malaria endemic area.

## Conclusions

In conclusion, the concentration of OPN is significantly higher in cord blood and infants living in a malaria-endemic area compared to OPN concentration in corresponding mothers. Infant OPN concentrations were independent of OPN concentration in corresponding mothers, even in cord blood, indicating that OPN is produced either by the fetus itself or in the placenta. In addition, OPN was associated with the presence of atypical memory B cells specific for *P. falciparum* malaria as well as BAFF, indicating a role in the naturally acquired immune response against malaria. *In vitro P. falciparum* inhibition assays did not support a direct inhibitory effect of OPN on parasite growth. Further studies of *P. falciparum* positive individuals with symptomatic malaria are warranted to advance understanding of the function of OPN in malaria.

## Methods

### Study design

The study was conducted from March 2012 to July 2013 at Kasangati Health Centre in Uganda. Kasangati is situated within a moderate malaria transmission area, with a peak transmission after the two main rainy seasons (February-March and September-October) every year. The cohort has previously been described in detail elsewhere [21]. In brief, healthy mother-infant pairs were randomly enrolled at birth and subsequently followed by the study clinic for three visits. Mothers were sampled at birth and after nine months. Infants were sampled at birth and at time points coinciding with the regular childhood vaccination schedule; 2.5 months, 6 months, and 9 months of age. None of the study individuals had any signs of severe infection at any point of sampling. During pregnancy, every pregnant woman took at least one or two doses of Fansidar (pyrimethamine plus sulfadoxine) intermittent preventive treatment and received a long-lasting insecticide mosquito bed net. At baby delivery, venous blood (5 mL) was collected into EDTA tubes from the mothers and the respective umbilical cord of the new-born infant. At follow-up days, blood (2 to 4 mL) from infants and 5 mL from their respective mothers were collected. The tubes were transported to the lab by motorbike. Upon arrival, peripheral blood mononuclear cells (PBMCs), cord blood mononuclear cells (CBMCs) and plasma were separated by density gradient centrifugation using Ficoll-hypaque (GE HealthCare Bio-Sciences AB, Sweden) and then stored in liquid nitrogen. Blood samples were transported to Sweden and thawed for analysis. All samples were handled in a similar manner. Out of the 131 original mother-infant pairs, 80 had enough volumes to perform measurements of OPN. Voluntary informed consent was obtained from all mothers participating in the study. The study was approved by the Makerere University, School of Medicine, Research and Ethics Committee, the Uganda National Council of Science and Technology (approval 2011-114) and Regionala Etikprövningsnämnden in Stockholm, Sweden (2014/478-32).

### Malaria diagnostics

All samples were tested by pLDH/HRP2 rapid diagnostic test strips (Combo Rapid Diagnostic Test of Premier Medical Corporation Limited, India) as described by Bharti et al. [81]. Thin and thick blood smears for all RDT positive samples were examined for malaria parasites, and the parasitemia was calculated according to the WHO guidelines [82].

### Osteopontin ELISA

The plasma concentration of OPN was measured by the Quantikine Human Osteopontin Immunoassay (R&D Systems, Abingdon, UK) according to the manufacturer’s instruction. In summary, 100 µL of assay diluent was added to each well followed by 50 µL of standard or plasma sample. Plasma samples were diluted 1:25 for the majority of samples (1:100 in case of plasma shortage). The plates were incubated for two hours, washed x4, resuspended with 200 µL of conjugate/well and incubated for 2 h, then washed x4 and 200 µL substrate solution was added and incubated for 30 min. After addition of 50 µL of stop solution/well the plate was read within 30 min. Assays were performed in duplicate on diluted samples and standards. To prevent batch/plate effects, longitudinal samples from the same mother-baby pairs and individuals were analyzed on the same plates. Plasma OPN concentrations were quantified using the in-kit standard curve ranging from 0.313 ng/mL to 20 ng/mL. Optical density was determined at 450 nm using a SPECTRA max340PC384 microplate reader. OPN concentrations were calculated using SoftMax Pro Software.

### *P. falciparum* schizont extract ELISA

Total *P. falciparum+* IgG and IgM in blood plasma were measured as previously described [21]. Briefly, microtiter plates were coated with schizont extract, blocked with 5 % skimmed milk (Sigma) for the IgG-assay and super block dry blend (Thermo Scientific) for the IgM-assay. Plates were incubated with peroxidase-conjugated goat anti-human IgG/IgM (Sigma) and bound antibody quantified using TMB (3,3′,5,5′-Tetramethylbenzidine) substrate (Promega). Optical density (OD) was read at 450 nm.

### Immunophenotyping of *P. falciparum* specific B cells

Immunophenotyping of *P. falciparum* was done according to the protocol described elsewhere [21]. Briefly, cryopreserved PBMCs were used together with Fc block and the following fluorochrome-conjugated monoclonal antibodies: CD19 PE-CF594-clone HIB19, CD20 V450-clone L27, CD27 PE-Cy7-clone M-T271 (all from BD), FcRL4 APC-clone 413D12 (Biolegend) and FITC-conjugated mouse anti-human IgG monoclonal antibody (BD Horizon). B cells specific for *P. falciparum* were identified utilizing carboxyl Quantum dots (Invitrogen) conjugated to extract of schizont- and trophozoite-stage parasites as described in detail by Lugaajju et al. [83]. The analysis was performed on a LSRII flow cytometer (Becton–Dickinson Immuno Cytometry Systems, San Jose, USA). To detect the proportion of B cells (defined as CD19+ cells) that were specific for *P. falciparum*, the cells were categorized as follows: IgG MBCs (CD19+CD20+CD27+FcRL4±IgG+), non-IgG+ MBCs (CD19+CD20+CD27+FcRL4±IgG−), naïve B cells (CD19+CD20+CD27−FcRL4±IgG−), plasma cells/blasts (CD19+CD20−CD27+FcRL4±IgG−), and atypical MBCs (CD19+CD20+CD27−FcRL4±IgG+). Data was processed using FLOWJO software (Tree Star Inc., San Carlos, and Ca, USA).

### *P. falciparum* culture and invasion assay with OPN

Details of the invasion inhibition method have been described elsewhere [43, 84]. Briefly, *in vitro* culture of *P. falciparum* FCR3S1.2 was maintained using O^+^ RBC at 4 % hematocrit in RPMI 1640-HEPES supplemented with 1 % Albumax II, 25 µg/mL gentamicin, 5 mM L-glutamine and 25 mM NaHCO_3_. The parasites were synchronized using 5 % D-sorbitol (Sigma) and all cultures were maintained in candle-light boxes at 37^°^ C. Human recombinant osteopontin (298 aa, Sigma-Aldrich) ranging from 0.2 µg/mL to 12,8 µg/mL was added to late trophozoite-stage parasite cultures of 1 % parasitemia and 1 % hematocrit and each OPN concentration was tested in duplicate (100 µL culture volume) in 96-well U-bottom plates. Inhibitory (100 µg heparin/mL) and growth (PBS) controls were run concurrently. Fresh culture medium (10 µL) was gently added to each well at about 48 h. The assay was stopped around 90 h by staining cells with acridine orange (10 µg/mL) and then fixed using 20 %/2 % formaldehyde/glutaraldehyde. Parasitemia was then determined using a flow cytometer (Accuri C6, BD) after cells had been washed and resuspended in PBS. The data was further analyzed using FLOWJO software (BD). This assay was repeated and the mean parasitemia obtained from four wells was used in the final analysis for each OPN concentration.

### Statistical analysis

Statistical analysis was performed using SPSS software, IBM SPSS Statistics for Macintosh Version 26 (IBM Corporation, Armonk, NY), R Statistical Software version 4.0.0 (Foundation for Statistical Computing, Vienna, Austria) and GraphPad Prism version 8.4.3 for macOS (GraphPad Software, San Diego, California USA). Differences of p < 0.05 were considered significant. Continuous variables were expressed as estimated means with corresponding confidence intervals or medians with interquartile ranges. Differences in OPN concentrations between groups were assessed by nonparametric Wilcoxon matched-pairs signed rank and by Kruskal-Wallis test for paired and unpaired samples, respectively. To take into account the repeated measurement nature of the data, linear mixed models were employed to model how OPN changed over time, with time points added as a fixed effect and individuals added as a random effect. Three models were used; two for infants and mothers separately, and one where the OPN concentrations in infants were adjusted for the OPN concentration in corresponding mothers at time of birth. For each time point, correlations between OPN concentrations in mothers or infants and corresponding mean proportions of various memory cells were estimated using Pearson correlations and correction for multiple comparisons using the Benjamini-Hochberg method. Multiple comparisons of mean parasitemias obtained from testing a range of OPN concentrations in invasion inhibition assays were carried out using ANOVA. All methods were performed in accordance with relevant guidelines and regulations.

## Supplementary Information

**Additional file 1.**

**Additional file 2.**

## Data Availability

All results are visible in the figures, and exact datasets used and/or analyzed during the current study are available from the corresponding author on reasonable request.

## Abbreviations

*P. falciparum*: *Plasmodium falciparum*
OPN: Osteopontin
BAFF: B cell activating factor
MBCs: Memory B cells
RBC: Red blood cells
*Pf+*: *Plasmodium falciparum* positive
PBS: Phosphate buffered saline
PBMC: Peripheral blood mononuclear cells

## References

[1] World Health Organization. World Malaria Report 2020 [Internet]. Geneva: World Health Organization; 2020. [cited 2021 May 05]. Available from: https://www.who.int/publications/i/item/9789240015791.

[2] Omer SA, Idress HE, Adam I, Abdelrahim M, Noureldein AN, Abdelrazig AM (2017). Placental malaria and its effect on pregnancy outcomes in Sudanese women from Blue Nile State. Malar J.

[3] Tako EA, Zhou A, Lohoue J, Leke R, Taylor DW, Leke RF (2005). Risk factors for placental malaria and its effect on pregnancy outcome in Yaounde, Cameroon. Am J Trop Med Hyg.

[4] De Beaudrap P, Turyakira E, White LJ, Nabasumba C, Tumwebaze B, Muehlenbachs A (2013). Impact of malaria during pregnancy on pregnancy outcomes in a Ugandan prospective cohort with intensive malaria screening and prompt treatment. Malar J.

[5] Ayres Pereira M, Mandel Clausen T, Pehrson C, Mao Y, Resende M, Daugaard M (2016). Placental Sequestration of Plasmodium falciparum Malaria Parasites Is Mediated by the Interaction Between VAR2CSA and Chondroitin Sulfate A on Syndecan-1. PLoS Pathog.

[6] Tran TM, Li S, Doumbo S, Doumtabe D, Huang CY, Dia S (2013). An intensive longitudinal cohort study of Malian children and adults reveals no evidence of acquired immunity to Plasmodium falciparum infection. Clin Infect Dis.

[7] Doolan DL, Dobaño C, Baird JK (2009). Acquired immunity to malaria. Clin Microbiol Rev.

[8] Kumar R, Loughland JR, Ng SS, Boyle MJ, Engwerda CR (2020). The regulation of CD4(+) T cells during malaria. Immunol Rev.

[9] Cockburn IA, Seder RA (2018). Malaria prevention: from immunological concepts to effective vaccines and protective antibodies. Nat Immunol.

[10] Cohen S, Mc GI, Carrington S (1961). Gamma-globulin and acquired immunity to human malaria. Nature.

[11] Banic DM, de Oliveira-Ferreira J, Pratt-Riccio LR, Conseil V, Goncalves D, Fialho RR (1998). Immune response and lack of immune response to Plasmodium falciparum P126 antigen and its amino-terminal repeat in malaria-infected humans. Am J Trop Med Hyg.

[12] Druilhe P, Spertini F, Soesoe D, Corradin G, Mejia P, Singh S (2005). A malaria vaccine that elicits in humans antibodies able to kill Plasmodium falciparum. PLoS Med.

[13] Roussilhon C, Oeuvray C, Muller-Graf C, Tall A, Rogier C, Trape JF (2007). Long-term clinical protection from falciparum malaria is strongly associated with IgG3 antibodies to merozoite surface protein 3. PLoS Med.

[14] Osier FH, Feng G, Boyle MJ, Langer C, Zhou J, Richards JS (2014). Opsonic phagocytosis of Plasmodium falciparum merozoites: mechanism in human immunity and a correlate of protection against malaria. BMC Med.

[15] Fried M, Nosten F, Brockman A, Brabin BJ, Duffy PE (1998). Maternal antibodies block malaria. Nature.

[16] Staalsoe T, Shulman CE, Bulmer JN, Kawuondo K, Marsh K, Hviid L (2004). Variant surface antigen-specific IgG and protection against clinical consequences of pregnancy-associated Plasmodium falciparum malaria. Lancet.

[17] Portugal S, Pierce SK, Crompton PD (2013). Young lives lost as B cells falter: what we are learning about antibody responses in malaria. J Immunol.

[18] Banic DM, Viana-Martins FS, De Souza JM, Peixoto TD, Daniel-Ribeiro C (1991). Polyclonal B-lymphocyte stimulation in human malaria and its association with ongoing parasitemia. Am J Trop Med Hyg.

[19] Asito AS, Moormann AM, Kiprotich C, Ng’ang’a ZW, Ploutz-Snyder R, Rochford R. Alterations on peripheral B cell subsets following an acute uncomplicated clinical malaria infection in children. Malar J. 2008;7:238. 10.1186/1475-2875-7-238 PMC2626599 19019204

[20] Daniel-Ribeiro CT, Zanini G (2000). Autoimmunity and malaria: what are they doing together?. Acta Trop.

[21] Lugaajju A, Reddy SB, Wahlgren M, Kironde F, Persson KEM (2017). Development of Plasmodium falciparum specific naive, atypical, memory and plasma B cells during infancy and in adults in an endemic area. Malar J.

[22] Zander RA, Butler NS (2013). Dysfunctional adaptive immunity during parasitic infections. Curr Immunol Rev.

[23] Ampomah P, Stevenson L, Ofori MF, Barfod L, Hviid L (2014). Kinetics of B cell responses to Plasmodium falciparum erythrocyte membrane protein 1 in Ghanaian women naturally exposed to malaria parasites. J Immunol.

[24] Clemente N, Raineri D, Cappellano G, Boggio E, Favero F, Soluri MF (2016). Osteopontin Bridging Innate and Adaptive Immunity in Autoimmune Diseases. J Immunol Res.

[25] Hao C, Cui Y, Owen S, Li W, Cheng S, Jiang WG (2017). Human osteopontin: Potential clinical applications in cancer (Review). Int J Mol Med.

[26] Lund SA, Giachelli CM, Scatena M (2009). The role of osteopontin in inflammatory processes. J Cell Commun Signal.

[27] Singh M, Ananthula S, Milhorn DM, Krishnaswamy G, Singh K (2007). Osteopontin: a novel inflammatory mediator of cardiovascular disease. Front Biosci.

[28] Rittling SR, Singh R (2015). Osteopontin in Immune-mediated Diseases. J Dent Res.

[29] Brown LF, Berse B, Van de Water L, Papadopoulos-Sergiou A, Perruzzi CA, Manseau EJ (1992). Expression and distribution of osteopontin in human tissues: widespread association with luminal epithelial surfaces. Mol Biol Cell.

[30] Ashkar S, Weber GF, Panoutsakopoulou V, Sanchirico ME, Jansson M, Zawaideh S (2000). Eta-1 (osteopontin): an early component of type-1 (cell-mediated) immunity. Science.

[31] Lampe MA, Patarca R, Iregui MV, Cantor H (1991). Polyclonal B cell activation by the Eta-1 cytokine and the development of systemic autoimmune disease. J Immunol.

[32] Iizuka J, Katagiri Y, Tada N, Murakami M, Ikeda T, Sato M (1998). Introduction of an osteopontin gene confers the increase in B1 cell population and the production of anti-DNA autoantibodies. Lab Invest.

[33] Kaleta B (2014). Role of osteopontin in systemic lupus erythematosus. Arch Immunol Ther Exp (Warsz).

[34] Frenzel DF, Borkner L, Scheurmann J, Singh K, Scharffetter-Kochanek K, Weiss JM (2015). Osteopontin deficiency affects imiquimod-induced psoriasis-like murine skin inflammation and lymphocyte distribution in skin, draining lymph nodes and spleen. Exp Dermatol.

[35] Husain-Krautter S, Kramer JM, Li W, Guo B, Rothstein TL (2015). The osteopontin transgenic mouse is a new model for Sjogren’s syndrome. Clin Immunol.

[36] Rothstein TL, Guo B (2009). Receptor crosstalk: reprogramming B cell receptor signalling to an alternate pathway results in expression and secretion of the autoimmunity-associated cytokine, osteopontin. J Intern Med.

[37] Naradikian MS, Perate AR, Cancro MP (2015). BAFF receptors and ligands create independent homeostatic niches for B cell subsets. Curr Opin Immunol.

[38] Treml JF, Hao Y, Stadanlick JE, Cancro MP (2009). The BLyS family: toward a molecular understanding of B cell homeostasis. Cell Biochem Biophys.

[39] Ma N, He Y, Xiao H, Han G, Chen G, Wang Y (2014). BAFF maintains T-cell survival by inducing OPN expression in B cells. Mol Immunol.

[40] Maeno Y, Nakazawa S, Dao le D, Van Tuan N, Giang ND, Van Hanh T (2006). Osteopontin is involved in Th1-mediated immunity against Plasmodium falciparum infection in a holoendemic malaria region in Vietnam. Acta Trop.

[41] Maeno Y, Nakazawa S, Yamamoto N, Shinzato M, Nagashima S, Tanaka K (2006). Osteopontin participates in Th1-mediated host resistance against nonlethal malaria parasite Plasmodium chabaudi chabaudi infection in mice. Infect Immun.

[42] Rönnberg C, Lugaajju A, Nyman A, Hammar U, Bottai M, Lautenbach MJ (2021). A longitudinal study of plasma BAFF levels in mothers and their infants in Uganda, and correlations with subsets of B cells. PLoS One.

[43] Persson KEM, Lee CT, Marsh K, Beeson JG (2006). Development and optimization of high-throughput methods to measure Plasmodium falciparum-specific growth inhibitory antibodies. J Clin Microbiol.

[44] Joung KE, Christou H, Park KH, Mantzoros CS (2014). Cord blood levels of osteopontin as a phenotype marker of gestational age and neonatal morbidities. Obesity (Silver Spring).

[45] Kirirabwa NS, Kimuli D, Nanziri C, Sama D, Ntudhu S, Okello DA (2019). A four-year trend in pulmonary bacteriologically confirmed tuberculosis case detection in Kampala-Uganda. BMC Pulm Med.

[46] Exum NG, Kibira SPS, Ssenyonga R, Nobili J, Shannon AK, Ssempebwa JC (2019). The prevalence of schistosomiasis in Uganda: A nationally representative population estimate to inform control programs and water and sanitation interventions. PLoS Negl Trop Dis.

[47] Stenczer B, Rigo J, Prohaszka Z, Derzsy Z, Lazar L, Mako V (2010). Plasma osteopontin concentrations in preeclampsia - is there an association with endothelial injury?. Clin Chem Lab Med.

[48] Schack L, Lange A, Kelsen J, Agnholt J, Christensen B, Petersen TE (2009). Considerable variation in the concentration of osteopontin in human milk, bovine milk, and infant formulas. J Dairy Sci.

[49] Sennels HP, Jacobsen S, Jensen T, Hansen MS, Ostergaard M, Nielsen HJ (2007). Biological variation and reference intervals for circulating osteopontin, osteoprotegerin, total soluble receptor activator of nuclear factor kappa B ligand and high-sensitivity C-reactive protein. Scand J Clin Lab Invest.

[50] Nourkami-Tutdibi N, Graf N, Beier R, Zemlin M, Tutdibi E (2020). Plasma levels of osteopontin from birth to adulthood. Pediatr Blood Cancer.

[51] Wu CY, Wu MS, Chiang EP, Wu CC, Chen YJ, Chen CJ (2007). Elevated plasma osteopontin associated with gastric cancer development, invasion and survival. Gut.

[52] Pereira TA, Syn WK, Amâncio FF, Cunha PH, Caporali JF, Trindade GV (2016). Osteopontin Is Upregulated in Human and Murine Acute Schistosomiasis Mansoni. PLoS Negl Trop Dis.

[53] Wirestam L, Enocsson H, Skogh T, Padyukov L, Jönsen A, Urowitz MB (2019). Osteopontin and Disease Activity in Patients with Recent-onset Systemic Lupus Erythematosus: Results from the SLICC Inception Cohort. J Rheumatol.

[54] Bruha R, Jachymova M, Petrtyl J, Dvorak K, Lenicek M, Urbanek P (2016). Osteopontin: A non-invasive parameter of portal hypertension and prognostic marker of cirrhosis. World J Gastroenterol.

[55] Li JJ, Li HY, Gu F (2014). Diagnostic significance of serum osteopontin level for pancreatic cancer: a meta-analysis. Genet Test Mol Biomarkers.

[56] Vordermark D, Said HM, Katzer A, Kuhnt T, Hänsgen G, Dunst J (2006). Plasma osteopontin levels in patients with head and neck cancer and cervix cancer are critically dependent on the choice of ELISA system. BMC Cancer.

[57] Denhardt DT, Guo X (1993). Osteopontin: a protein with diverse functions. Faseb j.

[58] Szulc P, Seeman E, Delmas PD (2000). Biochemical measurements of bone turnover in children and adolescents. Osteoporos Int.

[59] Kim JH, Skates SJ, Uede T, Wong KK, Schorge JO, Feltmate CM (2002). Osteopontin as a potential diagnostic biomarker for ovarian cancer. Jama.

[60] Chagan-Yasutan H, Saitoh H, Ashino Y, Arikawa T, Hirashima M, Li S (2009). Persistent elevation of plasma osteopontin levels in HIV patients despite highly active antiretroviral therapy. Tohoku J Exp Med.

[61] Li Q, Lifson JD, Duan L, Schacker TW, Reilly C, Carlis J (2005). Potential roles of follicular dendritic cell-associated osteopontin in lymphoid follicle pathology and repair and in B cell regulation in HIV-1 and SIV infection. J Infect Dis.

[62] Burdo TH, Ellis RJ, Fox HS (2008). Osteopontin is increased in HIV-associated dementia. J Infect Dis.

[63] Koguchi Y, Kawakami K, Uezu K, Fukushima K, Kon S, Maeda M (2003). High plasma osteopontin level and its relationship with interleukin-12-mediated type 1 T helper cell response in tuberculosis. Am J Respir Crit Care Med.

[64] Shiratori B, Leano S, Nakajima C, Chagan-Yasutan H, Niki T, Ashino Y (2014). Elevated OPN, IP-10, and neutrophilia in loop-mediated isothermal amplification confirmed tuberculosis patients. Mediators Inflamm.

[65] Hasibuan FM, Shiratori B, Senoputra MA, Chagan-Yasutan H, Koesoemadinata RC, Apriani L (2015). Evaluation of matricellular proteins in systemic and local immune response to Mycobacterium tuberculosis infection. Microbiol Immunol.

[66] Shete A, Bichare S, Pujari V, Virkar R, Thakar M, Ghate M (2020). Elevated Levels of Galectin-9 but Not Osteopontin in HIV and Tuberculosis Infections Indicate Their Roles in Detecting MTB Infection in HIV Infected Individuals. Front Microbiol.

[67] Agah E, Zardoui A, Saghazadeh A, Ahmadi M, Tafakhori A, Rezaei N (2018). Osteopontin (OPN) as a CSF and blood biomarker for multiple sclerosis: A systematic review and meta-analysis. PLoS One.

[68] Wei R, Wong JPC, Kwok HF (2017). Osteopontin -- a promising biomarker for cancer therapy. J Cancer.

[69] Abdelaziz Mohamed I, Gadeau AP, Hasan A, Abdulrahman N, Mraiche F. Osteopontin: A Promising Therapeutic Target in Cardiac Fibrosis. Cells. 2019;8(12). 10.3390/cells8121558 PMC6952988 31816901

[70] Wen Y, Jeong S, Xia Q, Kong X (2016). Role of Osteopontin in Liver Diseases. Int J Biol Sci.

[71] Sullivan RT, Kim CC, Fontana MF, Feeney ME, Jagannathan P, Boyle MJ (2015). FCRL5 Delineates Functionally Impaired Memory B Cells Associated with Plasmodium falciparum Exposure. PLoS Pathog.

[72] Weiss GE, Crompton PD, Li S, Walsh LA, Moir S, Traore B (2009). Atypical memory B cells are greatly expanded in individuals living in a malaria-endemic area. J Immunol.

[73] Rubtsova K, Rubtsov AV, Cancro MP, Marrack P, Age-Associated B, Cells (2015). A T-bet-Dependent Effector with Roles in Protective and Pathogenic Immunity. J Immunol.

[74] Kannel K, Alnek K, Vahter L, Gross-Paju K, Uibo R, Kisand KV (2015). Changes in Blood B Cell-Activating Factor (BAFF) Levels in Multiple Sclerosis: A Sign of Treatment Outcome. PLoS One.

[75] Vincent FB, Morand EF, Schneider P, Mackay F (2014). The BAFF/APRIL system in SLE pathogenesis. Nat Rev Rheumatol.

[76] Scholzen A, Teirlinck AC, Bijker EM, Roestenberg M, Hermsen CC, Hoffman SL (2014). BAFF and BAFF Receptor Levels Correlate with B Cell Subset Activation and Redistribution in Controlled Human Malaria Infection. The Journal of Immunology.

[77] Nduati E, Gwela A, Karanja H, Mugyenyi C, Langhorne J, Marsh K (2011). The Plasma Concentration of the B Cell Activating Factor Is Increased in Children With Acute Malaria. The Journal of Infectious Diseases.

[78] Sakai J, Akkoyunlu M (2017). The Role of BAFF System Molecules in Host Response to Pathogens. Clinical Microbiology Reviews.

[79] Cheng CW, Tang KT, Fang WF, Lin JD (2019). Synchronized expressions of serum osteopontin and B cell-activating factor in autoimmune thyroid disease. Eur J Clin Invest.

[80] Portugal S, Obeng-Adjei N, Moir S, Crompton PD, Pierce SK (2017). Atypical memory B cells in human chronic infectious diseases: An interim report. Cell Immunol.

[81] Bharti PK, Silawat N, Singh PP, Singh MP, Shukla M, Chand G (2008). The usefulness of a new rapid diagnostic test, the First Response Malaria Combo (pLDH/HRP2) card test, for malaria diagnosis in the forested belt of central India. Malar J.

[82] World Health Organization (2009). Bench aids for malaria microscopy.

[83] Lugaajju A, Reddy SB, Ronnberg C, Wahlgren M, Kironde F, Persson KEM (2015). Novel flow cytometry technique for detection of Plasmodium falciparum specific B-cells in humans: increased levels of specific B-cells in ongoing infection. Malar J.

[84] Tijani MK, Babalola OA, Odaibo AB, Anumudu CI, Asinobi AO, Morenikeji OA (2017). Acquisition, maintenance and adaptation of invasion inhibitory antibodies against Plasmodium falciparum invasion ligands involved in immune evasion. PLoS One.

