# Supplementary material for: Osteopontin and malaria: no direct effect on parasite growth, but correlation with P. falciparum-specific B cells and BAFF in a malaria endemic area
Source: BMC Microbiol. 2021 Nov 6;21:307. doi: 10.1186/s12866-021-02368-y (PMC8571855; doi:10.1186/s12866-021-02368-y)
Supplement: Supplementary file 1 — Additional file 1. [file 12866_2021_2368_MOESM1_ESM.pdf]

Supplementary Table 1.

Plasma OPN levels and parasitemia (number of infected RBC/µL of blood) for mothers and infants.

**Time course**	**Pat ID**	**Parasitemia**	**OPN concentration ng/mL**
Mother at birth			
	12	48	125
	32	16	291
	58	2760	95
Infant at 2.5 months			
	120	9650	270
Infant at 6 months			
	56	91 760	433
	118	22 624	290
	125	3840	70
Mother at 9 months			
	91	1520	92
	96	800	262
