# Supplementary material for: Osteopontin and malaria: no direct effect on parasite growth, but correlation with P. falciparum-specific B cells and BAFF in a malaria endemic area
Source: BMC Microbiol. 2021 Nov 6;21:307. doi: 10.1186/s12866-021-02368-y (PMC8571855; doi:10.1186/s12866-021-02368-y)
Supplement: Supplementary file 2 — Additional file 2. [file 12866_2021_2368_MOESM2_ESM.pdf]

Supplementary Figure 1. X-Y scatter plots showing the relationship between OPN and different B cell subsets and BAFF, significant by Pearson's correlation test with adjustment for multiple comparisons using the Benjamini-Hochberg method.

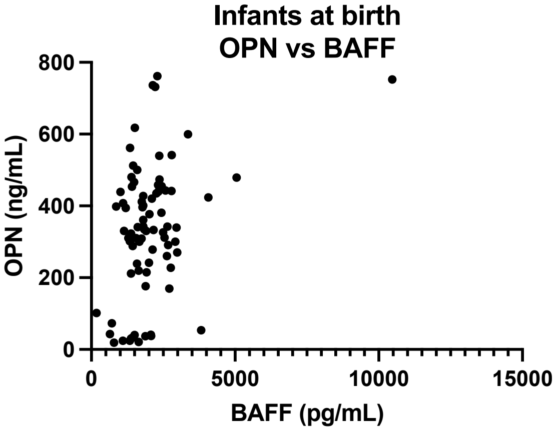

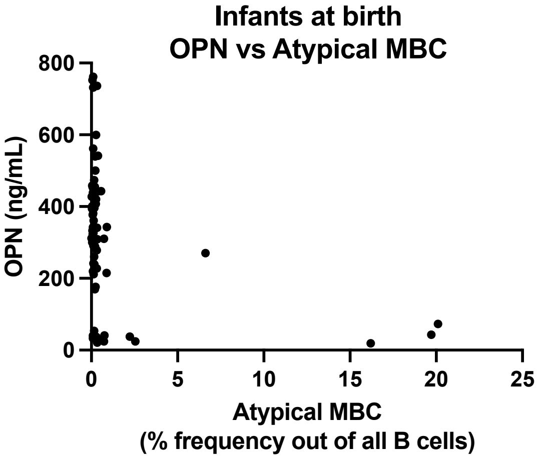

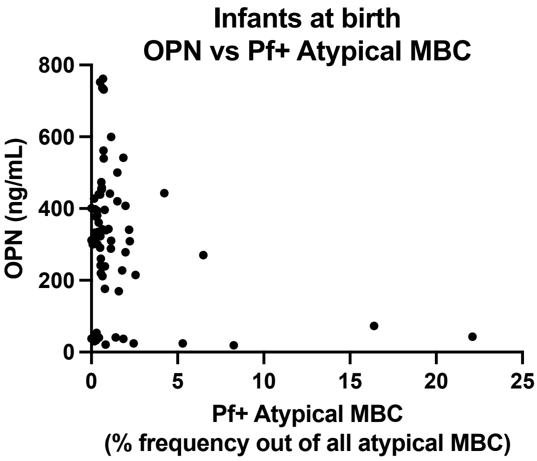

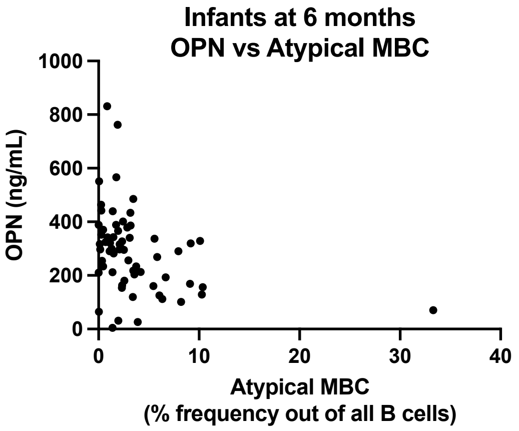
Infants at 6 months
OPN vs Atypical MBC
OPN (ng/mL)
1000
800
600
400
200
0
0
10
20
30
40
Atypical MBC
(% frequency out of all B cells)

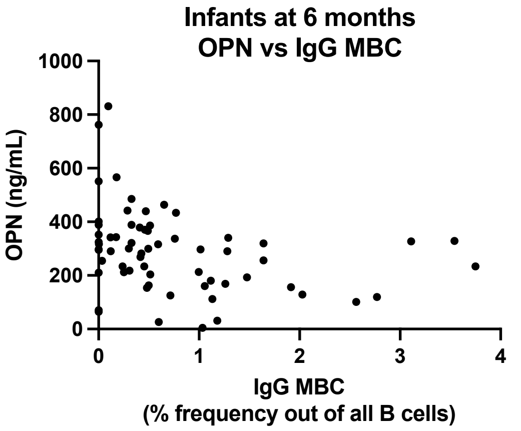
Infants at 6 months
OPN vs IgG MBC
OPN (ng/mL)
1000
800
600
400
200
0
0
1
2
3
4
IgG MBC
(% frequency out of all B cells)

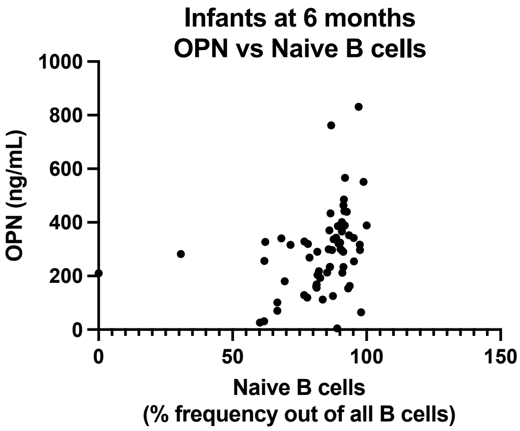
Infants at 6 months
OPN vs Naive B cells
OPN (ng/mL)
1000
800
600
400
200
0
0
50
100
150
Naive B cells
(% frequency out of all B cells)

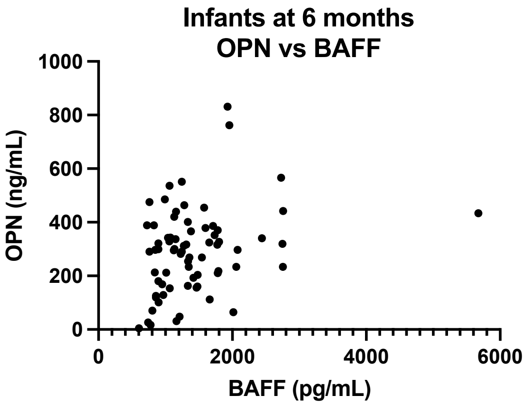
Infants at 6 months
OPN vs BAFF
OPN (ng/mL)
1000
800
600
400
200
0
0
2000
4000
6000
BAFF (pg/mL)

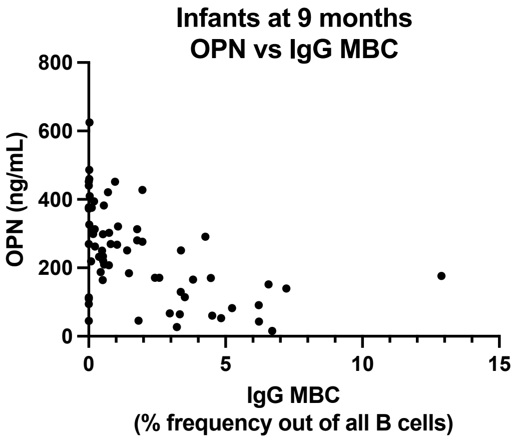
Infants at 9 months
OPN vs IgG MBC
OPN (ng/mL)
800
600
400
200
0
0
5
10
15
IgG MBC
(% frequency out of all B cells)

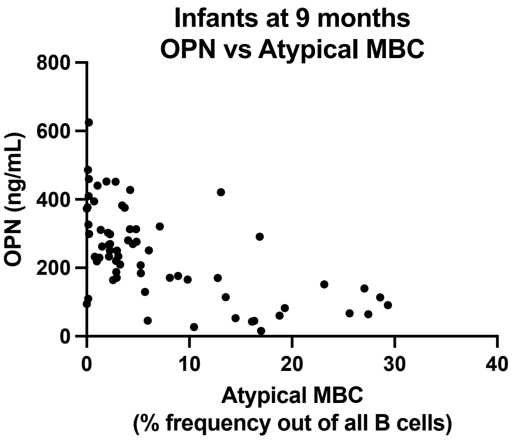
Infants at 9 months
OPN vs Atypical MBC
OPN (ng/mL)
800
600
400
200
0
0
10
20
30
40
Atypical MBC
(% frequency out of all B cells)

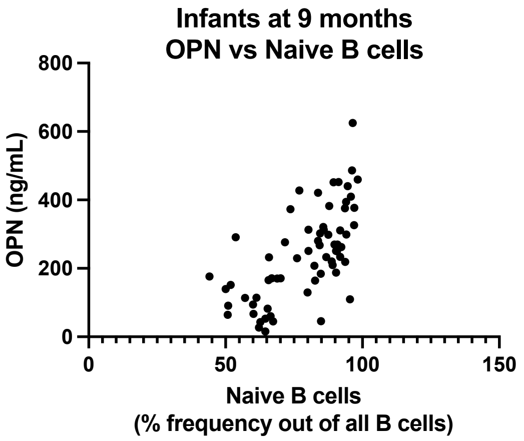
Infants at 9 months
OPN vs Naive B cells
OPN (ng/mL)
800
600
400
200
0
0
50
100
150
Naive B cells
(% frequency out of all B cells)

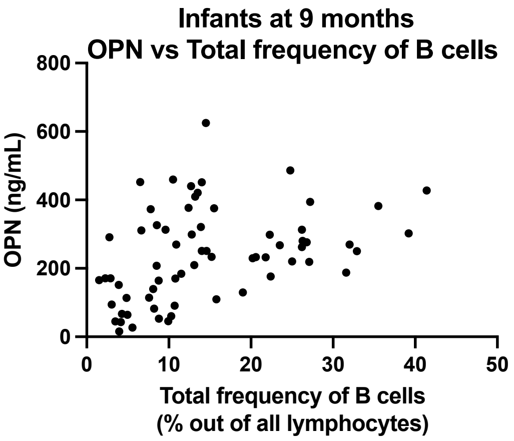
Infants at 9 months
OPN vs Total frequency of B cells
OPN (ng/mL)
800
600
400
200
0
0
10
20
30
40
50
Total frequency of B cells
(% out of all lymphocytes)

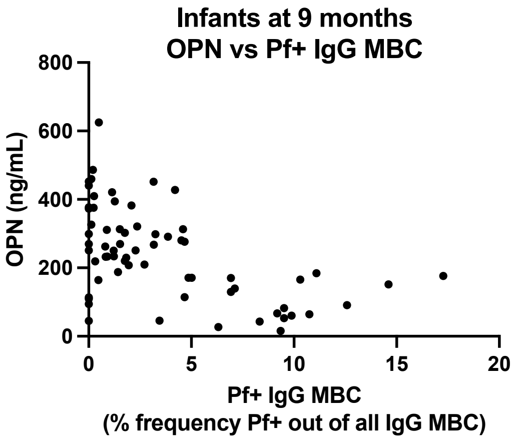

Infants at 9 months
OPN vs Pf+ IgG MBC
OPN (ng/mL)
800
600
400
200
0
0
5
10
15
20
Pf+ IgG MBC
(% frequency Pf+ out of all IgG MBC)

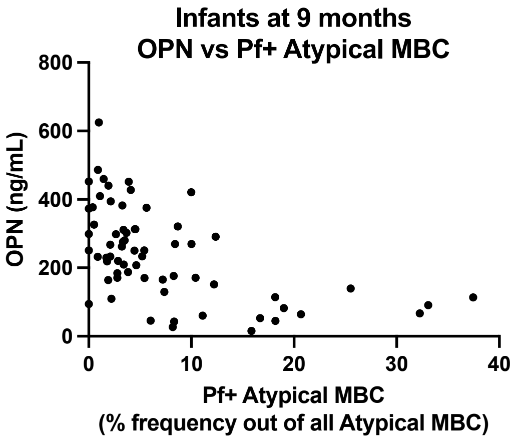

Infants at 9 months
OPN vs Pf+ Atypical MBC
OPN (ng/mL)
800
600
400
200
0
0
10
20
30
40
Pf+ Atypical MBC
(% frequency out of all Atypical MBC)

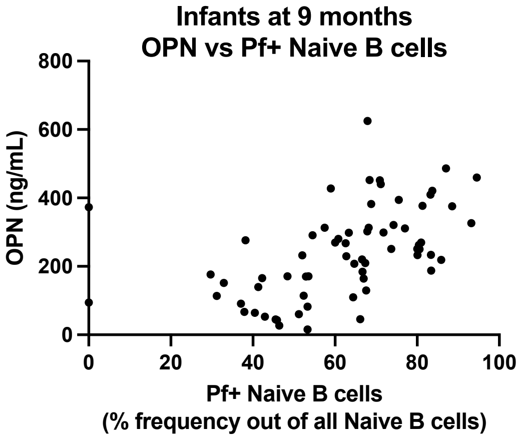

Infants at 9 months
OPN vs Pf+ Naive B cells
OPN (ng/mL)
800
600
400
200
0
0
20
40
60
80
100
Pf+ Naive B cells
(% frequency out of all Naive B cells)

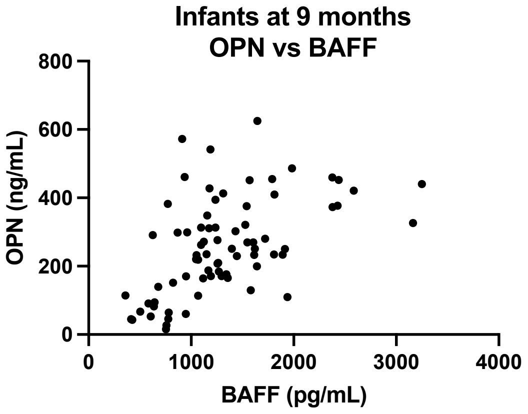

Infants at 9 months
OPN vs BAFF
OPN (ng/mL)
800
600
400
200
0
0
1000
2000
3000
4000
BAFF (pg/mL)

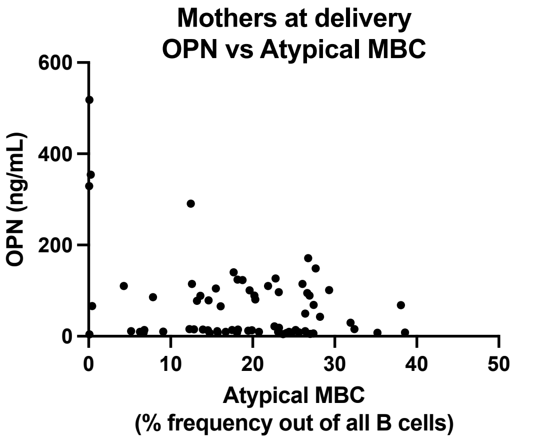
Mothers at delivery
OPN vs Atypical MBC
OPN (ng/mL)
600
400
200
0
0
10
20
30
40
50
Atypical MBC
(% frequency out of all B cells)

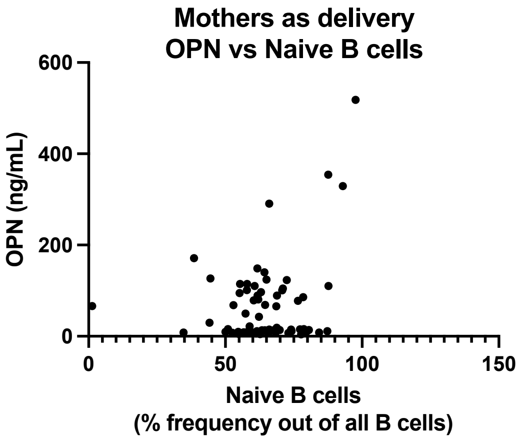
Mothers as delivery
OPN vs Naive B cells
OPN (ng/mL)
600
400
200
0
0
50
100
150
Naive B cells
(% frequency out of all B cells)

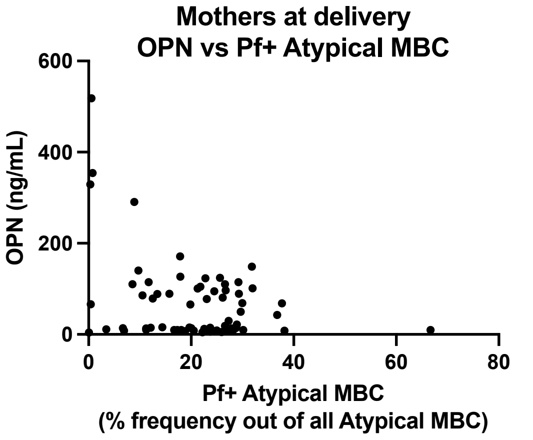
Mothers at delivery
OPN vs Pf+ Atypical MBC
OPN (ng/mL)
600
400
200
0
0
20
40
60
80
Pf+ Atypical MBC
(% frequency out of all Atypical MBC)

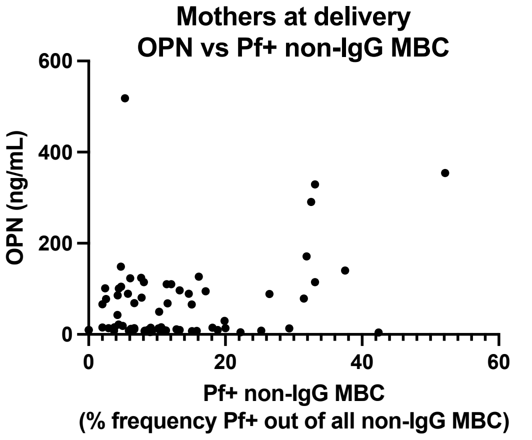
Mothers at delivery
OPN vs Pf+ non-IgG MBC
OPN (ng/mL)
600
400
200
0
0
20
40
60
Pf+ non-IgG MBC
(% frequency Pf+ out of all non-IgG MBC)

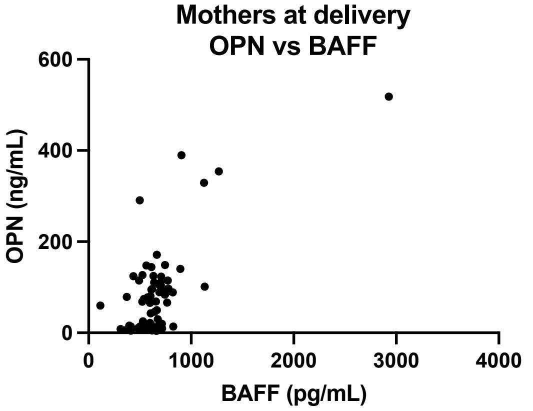
Mothers at delivery
OPN vs BAFF
OPN (ng/mL)
600
400
200
0
0
1000
2000
3000
4000
BAFF (pg/mL)
